# Common foods for boosting human immunity: A review

**DOI:** 10.1002/fsn3.3628

**Published:** 2023-08-18

**Authors:** Deo Narayan Singh, Jitendra Singh Bohra, Tej Pratap Dubey, Pushp Raj Shivahre, Ram Kumar Singh, Tejbal Singh, Deepak Kumar Jaiswal

**Affiliations:** ^1^ Department of Agronomy Udai Pratap Autonomous College Varanasi India; ^2^ Department of Agronomy, Institute of Agricultural Sciences Banaras Hindu University Varanasi India; ^3^ Council for Technical Education and Vocational Training (CTEVT) Bhaktapur Nepal; ^4^ Department of Animal Husbandry and Dairying Udai Pratap Autonomous College Varanasi India; ^5^ Institute of Pesticide Formulation Technology Gurugram India

**Keywords:** common food, functional food, human health, immunity

## Abstract

We are frequently exposed to potentially harmful microbes of various types on a daily basis. Our immune system is an amazing collection of unique organs and cells that defends us from hazardous germs as well as certain diseases. It plays a crucial role in protecting the body against external invaders, including bacteria, viruses, and parasites. Maintaining a healthy immune system requires consuming a balanced diet that provides a variety of macro‐ and micronutrients. By consuming sufficient amounts of water, minerals such as zinc and magnesium, micronutrients, herbs, and foods rich in vitamins C, D, and E, and adopting a healthy lifestyle, one can enhance their health and immunity, and prevent infections. This article provides a comprehensive review of the scientific literature on common foods known for their potential to boost human immunity. The review begins by discussing the various components of the immune system and their functions. It then delves into the current understanding of how nutrition can influence immune response, highlighting the importance of a well‐balanced diet in supporting optimal immune function. The article presents an extensive analysis of a range of common foods that have been studied for their immune‐boosting properties. These foods include fruits, vegetables, whole grains, and animal‐based foods. Each food category is explored in terms of its specific nutrients and bioactive compounds that contribute to immune support. Foods such as milk, eggs, fruits, leafy greens, and spices like onion, garlic, and turmeric contain beneficial compounds that can enhance the immune system's function, activate and inhibit immune cells, and interfere with multiple pathways that eventually lead to improved immune responses and defense. The available literature on the issue was accessed via online resources and evaluated thoroughly as a methodology for preparing this manuscript.

## Graphical abstract

Common foods contain bioactive compounds that boost immunity and provide essential nutrients. This article reviews immune‐boosting foods rich in nutrients, antioxidants, and bioactive compounds. These include citrus fruits, berries, leafy greens, garlic, ginger, yogurt, nuts, and seeds which offer unique benefits, from providing an array of bioactive compounds to boost human immunity.

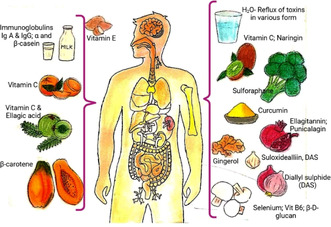

## 1 INTRODUCTION

The human body possesses multiple defense mechanisms against pathogenic invasion, and one such mechanism is the immune system—a sophisticated network of cells, tissues, and organs that collaborate to shield the human body from potential harm. Immunity can be categorized into two types: innate or nonspecific immunity, and acquired or specific immunity (Singh et al., 2022). Humans are born with some protective mechanism against harmful agents, which are ready to protect the body at a very short notice, constituting innate immunity, whereas acquired immunity is obtained through interaction with the invader and is peculiar to that invader. It is more specialized than innate immunity, and it complements and enhances innate immunity's protection (Coico & Sunshine, 2015). It is widely acknowledged that diet and nutrition have a significant impact on immune function. Our diet contains a variety of energy‐giving nutrients that serve as the foundation for building blocks, as well as nonnutrients that, in conjunction with essential nutrients, have distinct roles in regulating metabolism and other critical processes in our bodies. These processes include immune signaling, highlighting the importance of diet in modulating immunity (Pahwa & Sharan, 2022). Consuming a diet that is abundant in nutrients with immunomodulatory properties can aid in strengthening the immune system. Several foods have ingredients that help to keep our innate immunity (macrophages, NK cells, and neutrophils) as well as acquired immune system (T cells and B cells) in good working order (Vishwakarma et al., 2022). On the other hand, inadequate nutrition can impair immune system development and lead to immune incompetence, rendering the body more susceptible to infections (Cooper & Ma, 2017), allergies, and chronic inflammation (Albers et al., 2013). This underscores the importance of proper nutrition in maintaining optimal immune function. Dietary components have been demonstrated to prevent and treat a variety of diseases caused by immune system dysfunction, such as cancer and inflammatory conditions like atherosclerosis, cystic fibrosis, rheumatoid arthritis, bronchial asthma, and fibromyalgia (Bubnov et al., 2015). This highlights the potential therapeutic benefits of including certain foods in our diet to support immune function and combat disease.

Immunological cells require enough energy, with macro‐ and micronutrients acting as cofactors in the progress, articulation, and maintenance of the immune response. Protein‐rich foods aid immunoglobulin synthesis and possess antiviral properties (Ng et al., 2015; Norman et al., 2008; Schuetz et al., 2019). Therefore, individuals should take vegetables, nuts, legumes whole grains, and animal‐based meals as part of a normal diet. Certain foods, such as mushrooms, tomato, and chili pepper, and green vegetables like spinach and broccoli taken in regular meal help in building disease resistance in the body. Similarly, taking a low‐fat plant‐based diet may aid in strengthening the immune system (Mishra & Patel, 2020). Fiber can also help maintain a healthy body mass index (BMI), which is linked to better immunity (Rinninella et al., 2019). A plant‐based diet rich in vegetables, whole grains, and fruits has also been shown to lower inflammatory biomarkers (Soldati et al., 2018). Vitamins A, C, E, carotenoids, and flavonoids, for example, are widely accessible in the diet and act as antioxidants, scavenging oxidative free radicals (Waheed Janabi et al., 2020). Vitamin D supplementation was found to be safe in prevention of upper respiratory infections and influenza (Grant et al., 2020; Martineau et al., 2017). Naturally occurring functional food ingredients, such as carotenoids and flavonoids, have been identified as effective anti‐inflammatory agents and antioxidants that aid in the functioning of immune system (Kaur & Kapoor, 2001). Fruits and vegetables possess valuable health benefits that are associated with their anti‐inflammatory and antioxidant properties which are crucial in boosting the immune system and can be used as indicators for evaluating human health (Dangour et al., 2010).

The importance of a balanced diet in immune function has been well recognized (Maggini et al., 2008). Curcumin and ginger extract treatment resulted in faster healing in the skin of rats, which was discovered to be a unique way to improve the structure of the skin in rats. Curcumin reduces metastasis, invasion, cell proliferation, and angiogenesis in a variety of malignancies by inhibiting various protein signaling pathways (Bhagavathula et al., 2009). Iron deficiency anemia is treated with lemon and green leafy vegetables, which raise hemoglobin levels. It aids in the improvement of iron bioavailability in the blood (Shubham et al., 2020). Yogurt and banana, on the other hand, play an important role in the reciprocal advantages of probiotics and prebiotics. Prebiotics work as a fertilizer for good bacteria, whilst probiotics deliver good bacteria into the stomach (Natarajan et al., 2019).

Though the field of health and nutrition is most extensively studied one and several reviews have been published in this field, but most of them have focused on functional foods, and more importantly, almost all of the previous reviews have discussed nutrient components such as vitamins, minerals, etc. and their role in immunomodulation. This manuscript explores the relationship between food and immunity from a different angle, i.e., individual food item has been studied and discussed how these food items help in fighting illness and improving immunity with the perspective of common man while corroborating the facts with scientific evidence. Also, the field of nutrition and immune health is constantly evolving, with new studies and findings emerging regularly. A new review can provide updated information by incorporating the latest research and advancements in the field. It can offer a fresh perspective and ensure that readers have access to the most recent and relevant information on the subject. Considering the abovementioned facts, the present review has been designed with the objective to discuss the human immune system in brief and document the commonly available and easily accessible food items that help in maintaining healthy immune system and provide good resistance against diseases. This study will assist the general population in incorporating vital foods into their daily diets in order to strengthen and improve their immune system and overall health.

## 2 METHODOLOGY

This article aims to review common foods that have been reported to boost human immunity. The methodology involved conducting a comprehensive literature search to identify relevant studies and articles that examined the immunomodulatory properties of various foods. The selected articles were then analyzed to summarize the findings and provide insights into the effectiveness of these foods in enhancing human immune function. The review covers a wide range of foods, including fruits, vegetables, herbs, spices, and other dietary components. The findings presented in this article can serve as a valuable resource for individuals seeking to improve their immune systems through dietary choices. An extensive search of scientific databases (e.g., PubMed, Scopus, Web of Science, Google Scholar) using relevant keywords such as “immunity,” “immune system,” “immunomodulation,” “food,” and “nutrition” was done in order to find the relevant information. A wide range of studies published in peer‐reviewed journals including research papers, systematic reviews, and meta‐analyses that investigated the effects of specific foods on human immunity were reviewed.

## 3 IMMUNITY AND THE HUMAN IMMUNE SYSTEM

Immunity refers to the ability of the host to fight against pathogens, conferred by the immune system. Based on the immune response of the human body, immunity can be divided into two categories, viz., active immunity—in which the host's body itself produces the antibodies required for the immune response, and passive immunity—in which required antibodies are externally supplied to the body (Gangwar & Yadav, 2021). Passive immunity is short lived and has lesser importance in fight against the pathogens, whereas it is the active immunity which is mainly responsible for the immune responses in humans (Baxter, 2007).

**FIGURE 1:**
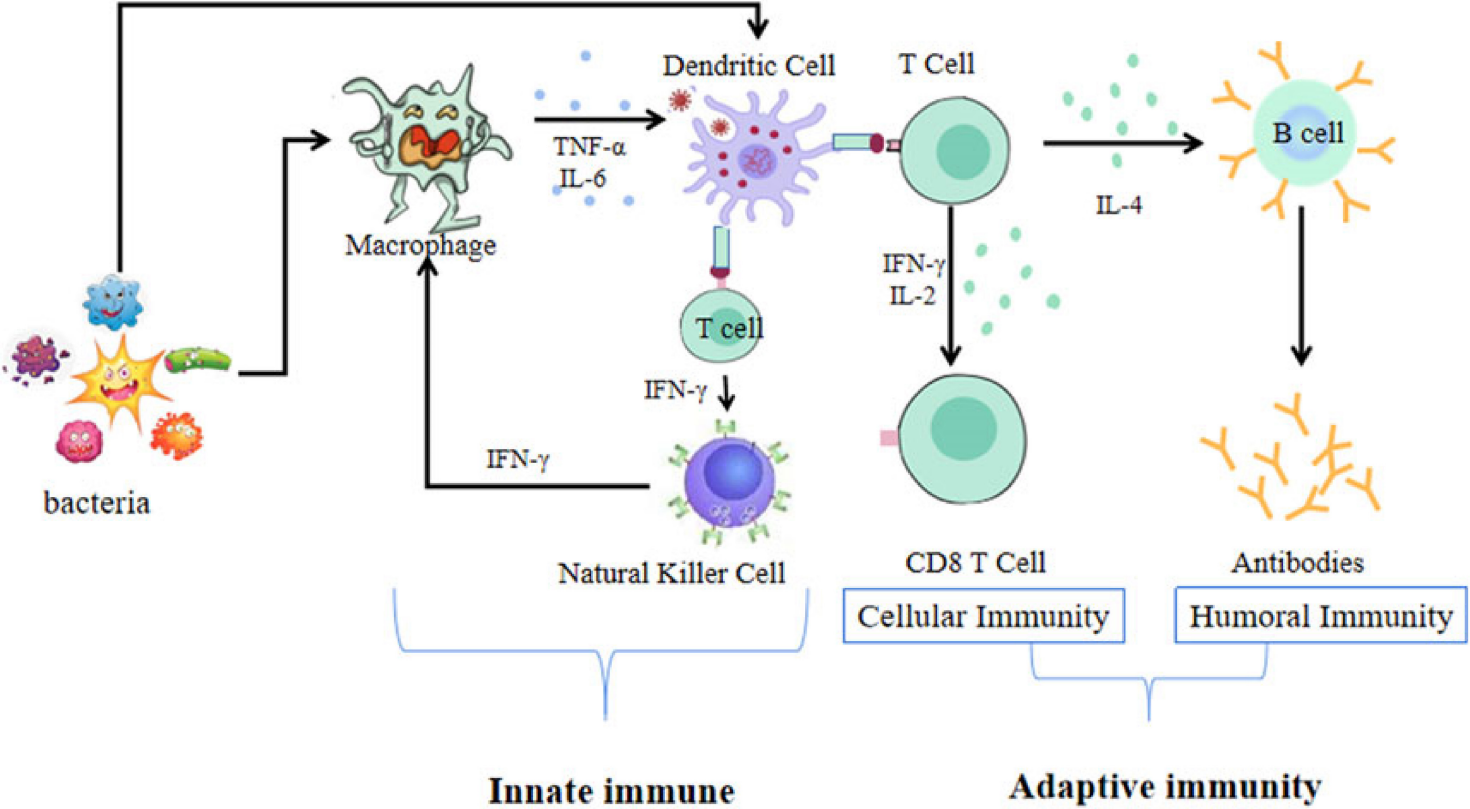
The human immune system (adopted from Jiang, 2019).

Active immunity is further divided into two categories, viz., innate immunity and acquired immunity based on its origin. Humans are born with innate immunity which mainly consists of four types of barriers: (1) physical barriers—like skin, and mucous coating the epithelium lining of respiratory system; (2) physiological barriers—acid in stomach, saliva in mouth, and tear from eyes; (3) cellular barriers—certain types of leukocytes (WBCs) of our body like polymorphonuclear leukocytes (MPNL—neutrophils), monocytes, and natural killers in the blood as well as macrophages in tissues act as cellular barrier by phagocytosis; and (4) complement proteins—a set of roughly 30 blood proteins that work together to protect the body against infection (Chaplin, 2010; Danilova, 2015; Marshall et al., 2018; Figure 1).

However, an individual develops the acquired immunity after birth, hence named. As one's body is exposed to pathogens or gets vaccinated, it develops a library of antibodies specific to each pathogen; this immunological memory remembers previous enemies and provides immunity against them in future attacks. As shown in Figure 1, the acquired immune responses are mainly governed by two types of lymphocytes, viz., B lymphocytes (B cells) and T lymphocytes (T cells). The role of B lymphocytes is the production of antibodies specific to each antigen produced by a pathogen. These antibodies, which come under the immunoglobulin family of substances has a multitude of functions in the immune system (Rich & Chaplin, 2019). The five main classes of immunoglobulins are IgA, IgD, IgE, IgG, and IgM. Each immunoglobulin class has unique chemical properties that allow it to perform specialized activities. IgA, for example, congregates in body fluids like tears and saliva, where it protects entry points; IgD is bound to B lymphocytes, helping them in initiating the immune response; IgE is responsible for allergies and protects against parasites; IgG works as a marker for the enemy and help the other cell in recognizing the enemy to deal with it; and IgM is specialized in killing bacteria (Schroeder & Cavacini, 2010). IgAs also aid in the management of commensal bacteria in mucosal regions and the protection of the body against infection (Hachimura et al., 2018). T lymphocytes are divided into three types: helper T cells, killer T cells, and suppressor T cells, each with its own purpose. Helper T cells are responsible for coordinating with other cells and stimulating B cells to generate additional antibodies. Killer T cells (also known as cytotoxic T lymphocytes) target other cells as their name implies. These aid in the detection and elimination of viruses by recognizing and targeting tiny fragments of viruses present on the surface of infected cells. At last, suppressor T cells provide the information to stop work by other T cells and B cells whenever the enemy is completely destroyed from the body (Cano & Lopera, 2013).

The various kinds of white blood cells mentioned above are kept in lymphoid organs located in various parts of the body like bone marrow, spleen, thymus, and lymph nodes. The principal lymphoid organ is the bone marrow, which produces all blood cells, including lymphocytes (Nigam & Knight, 2020). Lymphocytes that are not yet fully developed exit the bone marrow and migrate to the thymus, where they undergo a process of maturation to become functional T lymphocytes. Located between the lungs and below the neck, the thymus gland serves as the site for this process. On the other hand, positioned in the upper left abdomen, the spleen has a specialized physical structure that enables it to filter blood for infections and abnormal cells. Additionally, the spleen facilitates the formation of rare connections between antigen‐presenting cells (APCs) and matching lymphocytes (Lewis et al., 2019). Small glands known as lymph nodes are distributed throughout the body and play a crucial role in gathering and trapping germs and other foreign substances that enter the lymphatic and tissue fluids. Once antigens are trapped within the lymph nodes, they stimulate the lymphocytes present there, triggering an immune response (Liao & von der Weid, 2015; Mitra et al., 2022).

## 4 FOOD ITEMS AS IMMUNE BOOSTERS

Natural immune boosters are the products that we encounter in our daily activities. The first line of defense is always healthy lifestyle, which includes avoiding smoking, a diet rich in fruits and vegetables, exercise, regular sleep, and minimal stress (Monye & Adelowo, 2020). Humans have a long history of using natural immune boosters in their daily life, and it is gaining further momentum because of the increasing side effects of synthetic drugs (Tegegne & Kebede, 2022). Here, we discuss some of the commonly available food items and their role in immune system (Table 1).

**TABLE 1:** Common food items and their major bioactive compounds responsible for human immunity.

Common food items	Bioactive compound	Mechanism	References
Water	–	Reflux of toxins in various forms	Arshad et al. (2020) and Moeller et al. (2013)
Citrus fruits (*Citrus* spp.)	Vitamin C	Helps the epithelial barrier function, and boost the development of lymphocytes and phagocytes	Boretti and Banik (2020)
	Naringin	Anti‐inflammatory	Cheng et al. (2020)
Papaya (*Carica papaya* L.)	β‐carotene	Antioxidant, precursor of vitamin A, promotes lymphocyte and T‐cell proliferation	Farhan Aslam et al. (2017)
Mushrooms	Selenium	Antioxidant, anti‐inflammatory	Hoffmann and Berry (2008)
	Vitamin B6	Communication between cytokines and chemokines	Kunisawa and Kiyono (2013)
	β‐d‐glucan	Helps in the functioning of NK cells, T cells, macrophages, and B cells	Guo et al. (2012) and Lull et al. (2005)
Almonds (*Prunus amygdalus* L.)	Vitamin E	Antioxidant, T‐cell development	Chen et al. (2006)
Kiwi (*Actinidiaceae* spp. L.)	Vitamin C and polyphenols	Anti‐inflammatory	Baranowska‐Wójcik and Szwajgier (2019)
Aonla (Indian Gooseberry) (*Phyllanthus emblica* L.)	Vitamin C and Ellagic acid	Antioxidant	Kulkarni and Ghurghure (2018)
Pomegranate (*Punica granatum* L.)	Ellagitannins	Inducing apoptosis and inhibiting cell proliferation	Heber (2011)
	Punicalagin	Nuclear factor activation in activated T lymphocytes	Li et al. (2015)
Tea (*Camellia sinensis* L.)	Epigallocatechin gallate (EGCG)	Boosts the synthesis of immunoregulatory cytokines	Sultan et al. (2014)
	L‐theanine	Help in the generation of germ‐fighting chemicals in T cells	Chowdhury and Barooah (2020)
Broccoli (*Brassica oleracea* var. *italica*)	Sulforaphane (SFN)	Anti‐inflammatory and anticancer activities	Bessler and Djaldetti (2018)
Ginger (*Zingiber officinale* L.)	Gingerol	Antioxidant, analgesic, and anti‐inflammatory	Mashadi et al. (2013)
Garlic (*Allium sativum* L.)	Sulfoxide alliin, diallyl sulfide (DAS)	Stimulates natural killer cells, macrophages, dendritic cells, and eosinophils	Jiang (2019)
Onion (*Allium cepa* L.)	Diallyl sulfide, 146.2 Da; diallyl sulfoxide, 130.2 Da	Antioxidant, antibacterial, antiviral, antifungal, antimutagenic, anticarcinogenic antithrombotic, antihyperglycemic, prebiotic character, and immunosuppressive properties	Corzo‐martínez and Villamiel (2012) and Marefati et al. (2021)
Turmeric (*Curcuma longa* L.)	Curcumin	Modulates the function of immune cells like B cells, macrophages, monocytes, dendritic cells, and neutrophils	Srivastava et al. (2011)
Milk	Immunoglobulins IgA and IgG	Antibody synthesis, lymphocyte proliferation, and regulation of cytokines	Park and Nam (2015)
	α and β‐casein	Improve lymphocyte function	Marcone et al. (2017)
Egg	Phosvitin and ovotransferrin	Prevent the oxidation of lipids by metal chelation and free radicals scavenging	Jung et al. (2012)

*Source*: Adopted from various research and review papers (Chowdhury & Barooah, 2020; Field et al., 2002; Hachimura et al., 2018) on individual food items as cited against each of them.

### 4.1 Water

Drinking adequate water is important for a variety of reasons, one of them being reducing illness risk. Staying hydrated allows nutrients to reach all regions of the body and keep all bodily systems and organs functioning, potentially reducing the risk of illness (Rosinger, 2019). A sufficient quantity of water keeps the mucous membranes wet, reducing the risk of cold and flu. Drinking water aids in the oxygenation of cells, resulting in properly functioning systems. Well‐oxygenated cells are better placed in fighting against germs and other infectious agents than the cells having low oxygen. In addition, when someone is sick, the body loses a lot of water in the form of mucus, which is how infection‐causing microorganisms are removed from the body (Arshad et al., 2020; Moeller et al., 2013). Water plays a vital role in flushing out toxins from the body by carrying them through the kidneys and urinary tract. When the body is dehydrated, it can lead to a buildup of toxins in the bloodstream and other vital organs, which can lead to a weakened immune system. Drinking plenty of water throughout the day can help to prevent dehydration and promote the removal of toxins from the body. Staying hydrated is also critical for detoxification pathways, lymphatic drainage, and ensuring that foreign invaders and other waste items are flushed out. Muscle tension, headaches, decreased serotonin levels, and digestive problems can all be caused by dehydration (Quinn, 2020). Drinking more water can provide health benefits both directly, by increasing urine flow or dilution, and indirectly, by reducing the levels of osmotically induced vasopressin (AVP). Elevated levels of circulating AVP are associated with metabolic disorders, autosomal dominant polycystic kidney disease, and chronic kidney disease. Conversely, increased urine flow resulting from drinking more water can help prevent the formation of kidney stones and reduce the recurrence of urinary tract infections (Perrier et al., 2021).

## 5 PLANT‐BASED FOODS

### 5.1 Citrus fruits (*Citrus* spp.)

Grapefruit, oranges, clementines, tangerines, lemons, and limes are the popular citrus fruits. Vitamin C is an important micronutrient found in citrus fruits, which empowers the immune system by boosting both adaptive as well as innate immune cellular functioning. It helps the epithelial barrier function against infections (Boretti & Banik, 2020). Vitamin C may also boost the development of infection‐fighting white blood cells called lymphocytes and phagocytes, especially the differentiation and proliferation of B and T cells, two of the most important immune system players. It can also help in prevention and cure of respiratory and systemic infections. Vitamin C is also known to function as an antioxidant, assisting in the battle against free radicals, which damage and impair the ability of immune system to operate effectively (Carr & Maggini, 2017). Because the human body cannot generate or retain vitamin C, it is critical to take high‐quality vitamin C sources on a regular basis, especially while someone is unwell, as vitamin C levels may further get depleted. Apart from vitamin C, citrus fruits contain other compounds like carotenoids, folic acid, dietary fibers, potassium, selenium, and a variety of phytochemicals making them an effective natural agent to fight against cancer (Sidana et al., 2013). The flavonoids present in citrus fruits act as anti‐inflammatory and antioxidative agents that enhance the immune system by reducing inflammation and speedy recovery from disease. Naringin, a flavonoid found in citrus fruits, has been shown to have anti‐inflammatory effects on cytokines that cause inflammation (Cheng et al., 2020). In addition to naringin, lemons contain D‐Limonene which can help support the immune system. Furthermore, the antioxidant present in lemons can aid in protecting the eyes from age‐related damage such as macular degeneration, and prevention of several diseases such as cancer and cardiovascular ailments (Kandasamy & Shanmugapriya, 2015).

### 5.2 Papaya (C*arica papaya* L.)

Papaya is rich in retinol, thiamine, riboflavin, niacin, folic acid, iron, potassium, calcium, and fiber but low in calories (Deshpande et al., 2021). It contains carotenoids such as β‐carotene and lycopene, enzymes such as chymopapain and papain, and antioxidants like vitamin C, all of which have been shown to have health benefits in reducing the severity of illnesses such as rheumatoid arthritis, osteoarthritis, and asthma (Yogiraj et al., 2014). β‐carotene, which can be converted into vitamin A, functions as an antioxidant and can further enhance the immune system's response. Vitamin A has been shown to improve the body's immune response and support growth, reproduction, and the production of blood cells. Additionally, retinoic acid, a derivative of Vitamin A has been found to promote the proliferation of lymphocytes and T‐cells at the site of inflammation or infection in the stomach (Farhan Aslam et al., 2017). It is necessary for effective innate immune response. Vitamin A deficiency has been linked to a decrease in the number and activity of killer cells and eosinophils, as well as a reduction in oxidative burst and phagocytic capacity of neutrophils and macrophages (Amimo et al., 2022).

### 5.3 Kiwi (*Actinidia* species L.)

There are three types of kiwi fruits that are commonly used for commercial purposes: kiwi (*Actinidia deliciosa*), golden kiwi (*Actinidia chinensis*), and hardy kiwi (*Actinidia arguta*) (Torkashvand et al., 2017). Kiwis, like papayas, are rich in important nutrients such as potassium, vitamin C, carotenoids, dietary fiber, vitamin K, and antioxidants. The high levels of vitamin C and polyphenols found in kiwis have anti‐inflammatory effects that help to regulate the immune system and reduce the risk of flu (Baranowska‐Wójcik & Szwajgier, 2019). In vitro studies have also shown that golden kiwi can impact the immune system by regulating cells and cytokinesis (Fatima et al., 2020).

### 5.4 Pomegranate (*Punica granatum* L.)

Pomegranate juice has been found to suppress the development of dangerous germs such as *E. coli* O157:H7, Listeria, Shigella, Clostridium, Yersinia, Salmonella, and *Staphylococcus aureus* (Bialonska et al., 2009; Howell & D'Souza, 2013). It has also been demonstrated to have antiviral properties, making it useful in fighting against viruses like flu. Pomegranate juice also promotes the growth of healthy gut flora, such as Bifidobacterium and *Lactobacillus*, which can significantly boost the immune system (Li et al., 2015). Pomegranate consumption reduces platelet aggregation, which is a major risk factor for cardiovascular disease. It also aids in the promotion of healthy digestion, bowel movement, weight loss, and body immunity (Kandasamy & Shanmugapriya, 2015).

### 5.5 Aonla (Indian gooseberry) (*Phyllanthus emblica* L.)

Aonla is a fruit that is abundant in vitamin C, flavonoids, and antioxidants and has been found to have immunomodulatory and anti‐inflammatory properties. Ellagic acid, which is present in aonla, is a potent antioxidant (Kulkarni & Ghurghure, 2018). Aonla has also been shown to promote natural killer cell activity and antibody‐dependent cell cytotoxicity. These properties make aonla a promising candidate for the prevention and treatment of cancer, due to its potential chemomodulatory, neuromodulatory, free radical scavenging, chemo‐preventive effects, antioxidant, antimutagenic, anti‐inflammatory, and immunomodulatory properties (Chu et al., 2022; Siddique et al., 2021). Aonla is rich in vitamin C, flavonoids, and antioxidants and has shown immunomodulatory activities and anti‐inflammation response. Ellagic acid found in aonla is a powerful antioxidant (Dasaroju & Gottumukkala, 2014).

### 5.6 Almonds (*Prunus amygdalus* L.)

Almonds are a rich source of vitamin E, which is essential, along with vitamin C, for maintaining a strong immune system. Vitamin E is a potent antioxidant that plays a critical role in supporting healthy immune function. In addition to vitamin E, almonds also contain monounsaturated and polyunsaturated fats, flavonoids like kaempferol, catechin, epicatechin, and isorhamnetin, as well as plant sterols. These components are key factors in almonds' ability to impact immunological and inflammatory processes (Burns et al., 2016). Almonds have an anti‐inflammatory effect on cardiovascular diseases, and the presence of vitamin E in the body acts as an antioxidant. Vitamin E is vital for T‐cell development as well (Chen et al., 2006).

### 5.7 Broccoli (*Brassica oleracea* var. *italica*)

Broccoli is a green vegetable with its fleshy stalk and large flowering head being consumed worldwide in various forms as cooked vegetable, salad, soup, etc. With its abundance of vitamins A, C, and E, fiber, and numerous antioxidants, broccoli is considered to be one of the healthiest vegetables available for consumption. Broccoli's beneficial effects on human health are owing to its high quantity of minerals, vitamins, and isothiocyanates, the most significant of which is sulforaphane (SFN). Anti‐inflammatory and anticancer activities have been demonstrated for SFN (Bessler & Djaldetti, 2018). Consumption of least cooked or raw broccoli is advised to preserve its potential. Steaming is also one of the best ways to cook food items while keeping their nutritional qualities intact (Yuan et al., 2009). The health benefits of broccoli go beyond its nutritional value, as it also contains various phytochemicals such as polyphenols, glucosinolates, and their derivatives, including isorhamnetin, quercetin glucosides, and kaempferol. These compounds contribute to the vegetable's antioxidant and anticancer properties, making it a favorite food among health‐conscious individuals. In addition, numerous epidemiological studies have confirmed the dietary and therapeutic advantages of broccoli, including its ability to modulate immunity, support detoxification, promote eye and bone health, and exhibit antimicrobial and antioxidant properties (Nagraj et al., 2020).

### 5.8 Ginger (*Zingiber officinale* Roscoe)

Ginger (*Zingiber officinale* Roscoe) is a popular dietary condiment that is utilized in various cuisines across the globe. The rhizomes of ginger are the source of oleoresin, which contains several bioactive components, including gingerol, the primary pungent ingredient believed to have remarkable pharmacological and physiological effects. Ginger is known for its ability to improve appetite, enhance digestion, act as an anticold agent, and provide analgesic and anti‐inflammatory benefits (Fatima et al., 2020). Red ginger, which contains bioactive compounds that are more potent than those found in normal ginger, has demonstrated promising results as an immunomodulator in the treatment of psoriasis. Studies have shown that red ginger can impact the activity of T lymphocytes (Suciyati & Adnyana, 2017). In addition to its immunomodulatory properties, ginger has been shown to have potential benefits in reducing chronic pain and lowering cholesterol levels (Lakhan et al., 2015; Pourmasoumi et al., 2018). Excessive production of reactive oxygen species (ROS) or free radicals during metabolism exceeds a biological system's antioxidant capacity, resulting in oxidative stress, which is implicated in heart disease, cancer, neurological illnesses, and the aging process. Gingers' bioactive compounds, including gingerols, have been found to have antioxidant action in a variety of modules (Mashadi et al., 2013). Increased consumption of antioxidant‐rich foods and beverages, such as ginger shots, may aid in the fight against inflammation and the maintenance of a healthy immune system (Renuka & Muralidharan, 2017).

### 5.9 Garlic (*Allium sativum* L.)

Garlic has been used as a popular herbal remedy in traditional medicine since time immemorial. A large number of biologically active sulfur‐containing compounds, such as sulfoxides, proteins, and polyphenols in freshly crushed garlic have been found to possess antiviral properties and enhance the immune system (Anywar et al., 2020; Sahoo & Banik, 2018). Garlic has been shown to possess immunostimulatory properties that may be useful in therapeutic applications. It is able to stimulate both innate and specific cell immunity through the activation of natural killer cells, lymphocytes, macrophages, dendritic cells, and eosinophils. These immune cells are known to improve the functioning of the immune system, thereby potentially reducing the risk of certain diseases. Garlic contains sulfoxide alliin, which is converted to allicin when crushed or chewed. Additionally, garlic's bioactive component diallyl sulfide (DAS) can block inflammatory factors such as reactive oxygen species (ROS), NF‐kB (nuclear factor kappa‐light‐chain‐enhancer of activated B cells), and cyclooxygenase‐2 expression via the NF‐kB pathway (Elengoe, 2020). This pathway is crucial for cytokine synthesis and cell survival, and blocking it may help to reduce inflammation. Garlic has demonstrated antithrombotic action and reduced platelet aggregation in humans in an in vitro study (Jiang, 2019). Its components have been demonstrated to have a range of immunomodulatory effects on leukocyte cytokine production (Arreola et al., 2015). Garlic consumption improves hematological characteristics, such as total white blood cell (WBC) count, as well as homeostatic parameters. Garlic consumption may further promote the generation and release of nitric oxide (NO), which is responsible for increased interferon‐alpha release in humans, which is effective against viral and proliferative illnesses (Sultan et al., 2014).

### 5.10 Onion (*Allium cepa* L.)

Onion (*Allium cepa* L.) is a commonly used ingredient in Indian cuisine and is one of the most widely cultivated and consumed vegetables around the world. Its use dates back to ancient Egypt, where it was valued for its antibacterial, anti‐inflammatory, and other medicinal properties (Marefati et al., 2021). The health benefits of onions are attributed to their bioactive components, including organosulfur compounds such as diallyl sulfide (146.2 Da) and diallyl sulfoxide (130.2 Da), proteins, peptides, and flavonoids, particularly quercetin derivatives. These compounds give onions their antioxidant, antibacterial, antiviral, antifungal, anticarcinogenic, anti‐inflammatory, and antimutagenic properties (Corzo‐martínez & Villamiel, 2012). Onion fructooligosaccharides (FOS) have strong mitogenic and phagocytic activity, suggesting a possible role in therapeutic immunomodulation (Kumar et al., 2015).

### 5.11 Turmeric (*Curcuma longa* L.)

Turmeric, a bright yellow and bitter spice commonly found in curries, has been used as an anti‐inflammatory agent to treat osteoarthritis and rheumatoid arthritis. Its active ingredient, curcumin, has been found to help reduce exercise‐induced muscle injury due to its high levels of yellow pigment (curcumin). Studies on animals have also shown that curcumin can boost immunity and possess antiviral properties (McFarlin et al., 2016). Curcumin, the active compound in turmeric, has been found to have a regulatory effect on various biological activities, signal transducers, transcription factors, mitogen‐activated protein kinase, cytokine release, and receptors on different immune cell types (Srivastava et al., 2011). It has been shown to modulate the function of immune cells such as B cells, dendritic cells, monocytes, macrophages, and neutrophils, ultimately affecting innate and adaptive immunity in pathological conditions. Curcumin is also known for its antioxidant properties, acting as a scavenger of oxygen free radicals, protecting hemoglobin from oxidation, and interfering with the replication of microbes and viruses (Catanzaro et al., 2018; Momtazi‐Borojeni et al., 2018; Rathaur et al., 2012).

### 5.12 Tea (*Camellia sinensis* L.)

Flavonoids, a type of antioxidant, are abundant in both green and black teas. Green tea contains epigallocatechin gallate (EGCG), an antioxidant that improves immunological function. EGCG boosts the synthesis of immunoregulatory cytokines, lowers the risk of various diseases, reduces inflammation, and protects cells from harm (Sultan et al., 2014). The fermentation process used to manufacture black tea removes a considerable percentage of the EGCG, whereas it is preserved in green teas as green tea, is steamed rather than fermented, which keeps the EGCG intact. Green tea is also rich in L‐theanine, an amino acid, which may help in the creation of germ‐fighting chemicals in T cells (Chowdhury & Barooah, 2020; Williams et al., 2020). Tea preparations possess antioxidant properties and contain phenolic compounds, such as Thearubigins and Theaflavins, which have been shown to have potential therapeutic effects against cancer, cardiovascular disease, and inflammation. These compounds have been recognized for their ability to act as antioxidants and provide various health benefits (Hayat et al., 2015). Unlike traditional tea made from *Camellia sinensis* leaves, herbal teas are a blend of various dried plant parts such as fruits, flowers, seeds, nuts, barks, and grasses, which may include chamomile, cinnamon, ginseng, ginger root, cardamom, parsley, and cloves, among others. These ingredients possess immune‐enhancing properties that can be beneficial to our health, including anti‐inflammatory, antiviral, antitumor, antioxidant, and antibacterial properties (Poswal et al., 2019).

### 5.13 Mushrooms

Mushrooms are edible fungi containing considerable quantities of selenium as well as B vitamins such as niacin and riboflavin, which play an important role in supporting the human immune system. Selenium, in particular, functions as an antioxidant and can help to reduce oxidative stress and inflammation, while also enhancing immune function (Hoffmann & Berry, 2008). Vitamin B6 aids in the communication between cytokines and chemokines, as well as improving immune response to increased antibody production (Kunisawa & Kiyono, 2013). A lack of vitamin B6 inhibits lymphocyte development and proliferation, antibody production, and T‐cell function (Rail & Meydani, 1993). Mushrooms contain a variety of bioactive compounds that contribute to their immune‐modulating properties, such as polysaccharides like β‐d‐glucan, polysaccharide–peptide/protein complexes, proteins, proteoglycans, and triterpenoids. Specifically, β‐d‐glucan extracted from mushrooms has been shown to stimulate the immune response of NK cells, B cells, T cells, and macrophages (Guo et al., 2012; Lull et al., 2005). They are also said to have anticholesterol, antiallergic, anticancer, and antitumor properties (Wani et al., 2010). Mushrooms have a variety of impacts, including the capacity to boost cytokine production, tiny, soluble proteins that function as intracellular mediators (Guggenheim et al., 2014).

## 6 ANIMAL‐SOURCED FOODS

Animal‐sourced foods (ASF) are high in calories and a good source of high‐quality, easily digested protein. The proteins in these foods are considered to be of the best quality and easily accessible by the body as they include a complete set of essential amino acids required for the human body. Animal‐based meals are also a good source of micronutrients. Mild‐to‐moderate protein‐energy malnutrition (PEM) is widespread in underdeveloped countries. Malnutrition is especially dangerous for children, as it relates to stunted growth, impaired mental development, and disease. Furthermore, the synergistic associations between PEM, infection, and immunological function are well established (Neumann et al., 2002). Considering this, the inclusion of animal‐based foods like milk, meat, eggs, and fish all contribute to a well‐balanced human diet, particularly in terms of immunity.

### 6.1 Milk

Milk is a rich source of essential nutrients, such as vitamins, minerals, and specialized proteins, which are crucial for maintaining good health (Bhat & Bhat, 2011). Its immunological properties have been recognized for a long time; prolactin, a hormone present in milk, can enhance immune development by promoting the movement of lymphocytes and thymocytes. Milk also contains immunoglobulins, such as IgA and IgG, that can modulate the humoral immune response. In addition, peptides and protein hydrolysates obtained from milk's caseins and major whey proteins have immunomodulatory effects, such as increasing lymphocyte proliferation, regulating cytokines, and stimulating antibody synthesis (Li et al., 2014; Park & Nam, 2015). Milk proteins (casein and whey) and milk fat constitute the majority of bovine milk's immunomodulatory properties. Incorporating whey protein and α‐lactalbumin into the diet improves lymphocyte function, while also improving the responsiveness of spleen‐generated lymphocytes to T‐cell mitogens. Similarly, lactoferrin (LF) and casenoglycopeptides (CGP) derived from K‐casein have been proven to improve lymphocyte function. In vitro experiments have shown that peptides generated from the enzymatic cleavage of α and β‐casein can also improve human lymphocyte activity (Ha et al., 2021; Marcone et al., 2017; Xue et al., 2023).

Fukushima et al. (2007) found improved blood cell phagocytic activity in elderly people when fermented milk was added to their diet. In addition to altering lymphocyte activity, it has been proven that dietary milk proteins regulate antibody responses. It has been observed that the use of α‐lactalbumin, α‐lactalbumin hydrolysate, and whole whey protein concentrate increases antibody production against foreign antigens (Bounous et al., 1981). Golden milk, a milk‐based drink (half a teaspoon of turmeric powder in 150‐mL hot milk) is supposed to increase immunity when consumed once or twice a day. Curcumin, the main ingredient in turmeric (*Curcuma longa* L.), regulates the synthesis of cytokines, especially the interleukin‐1, interleukin‐6, and tumor necrosis factor‐α (Sharifi‐Rad et al., 2020).

β‐Carotene, an antioxidant and precursor of vitamin A found in milk and can have several beneficial effects on the immune system. It can protect phagocytic cells from damage caused by oxidation, enhance the activity of effector T cells, and increase the ability of macrophages, cytotoxic T cells, and natural killer cells to eliminate cancerous cells. Vitamin A, which is derived from β‐Carotene, can modify the function and integrity of epithelial tissues, lymphoid mass, and specific as well as nonspecific immunity, thereby potentially reducing the risk of infections (Field et al., 2002).

### 6.2 Yogurt and curd

Yogurt is a product made from milk that undergoes fermentation by lactic acid bacteria (LAB) species which commonly include *Lactobacillus bulgaricus* and *Streptococcus thermophilus*. While both milk and yogurt have similar mineral compositions, certain minerals, such as calcium, are more easily absorbed from yogurt than milk. Additionally, yogurt contains less lactose and more lactic acid, peptides, galactose, free fatty acids, and free amino acids than milk (Meydani & Ha, 2000). Numerous studies have demonstrated that the therapeutic benefits of LAB and yogurt, such as their ability to boost the immune system, are largely attributable to alterations in the microecology of the gastrointestinal tract. The consumption of yogurt can increase the population of LAB in the intestines, which can help to suppress the growth of harmful bacteria, leading to a decrease in infections and an increase in anticarcinogenic effects (Ahmad & Ghosh, 2020). The extent to which LAB can stimulate the immune system also depends on their proximity to lymphoid tissues during their temporary colonization of the intestinal lumen (Meydani & Ha, 2000).

The consumption of curd has been shown to have a positive impact on human health, particularly by enhancing the immune system. Curd can increase natural immunity by stimulating both mucosal and systemic host immunity. This occurs through the activation of macrophages, elevation of immunoglobulin levels, and enhancement of natural killer cell activity and cytokine production within the body (Das et al., 2019). The regular consumption of yogurt can decrease the risk of cardiovascular diseases, chronic kidney diseases, and diabetes while simultaneously boosting the immune system of the host (Lisko et al., 2017).

### 6.3 Egg

Eggs obtained from domestic poultry birds are a cheap and widely accessible food in many regions of the world. These are rich sources of vitamins and minerals; however, there has been considerable debate in the past regarding whether eggs are healthy or not, particularly when it comes to cholesterol. Eggs are a good source of protein, which is crucial for maintaining and repairing muscles and other bodily structures. Additionally, eggs contain vitamins and minerals that are essential for proper brain and neurological function. High levels of vitamin A, vitamin B‐12, and selenium in eggs help strengthen the immune system (Puglisi & Fernandez, 2022). Choline, found in eggs, can assist in breaking down homocysteine, an amino acid linked to heart disease. Finally, the antioxidants lutein and zeaxanthin in eggs help prevent age‐related blindness caused by macular degeneration (Puglisi & Fernandez, 2022).

Eggs are an excellent source of antioxidants as well as high‐quality proteins that are comparable to those found in breast milk. The antioxidants (phosvitin and ovotransferrin) present in eggs prevent the oxidation of lipids by metal chelation and arrest free radicals (Jung et al., 2012). Other vitamins included in eggs help to maintain strong vision. Ovomucin, ovotransferrin, ovalbumin, avidin, and lysozyme are the major bioactive components of egg white having antibacterial activity. These proteins have antibacterial and immunomodulatory effects through inflammatory pathways (Batiha et al., 2021). Therefore, including eggs in normal diet may be a wise option to have a nutritious and healthy diet, especially in underdeveloped regions, where high value proteinaceous foods like pulses and milk are not accessible to common people.

## 7 CONCLUSION

**FIGURE 2:**
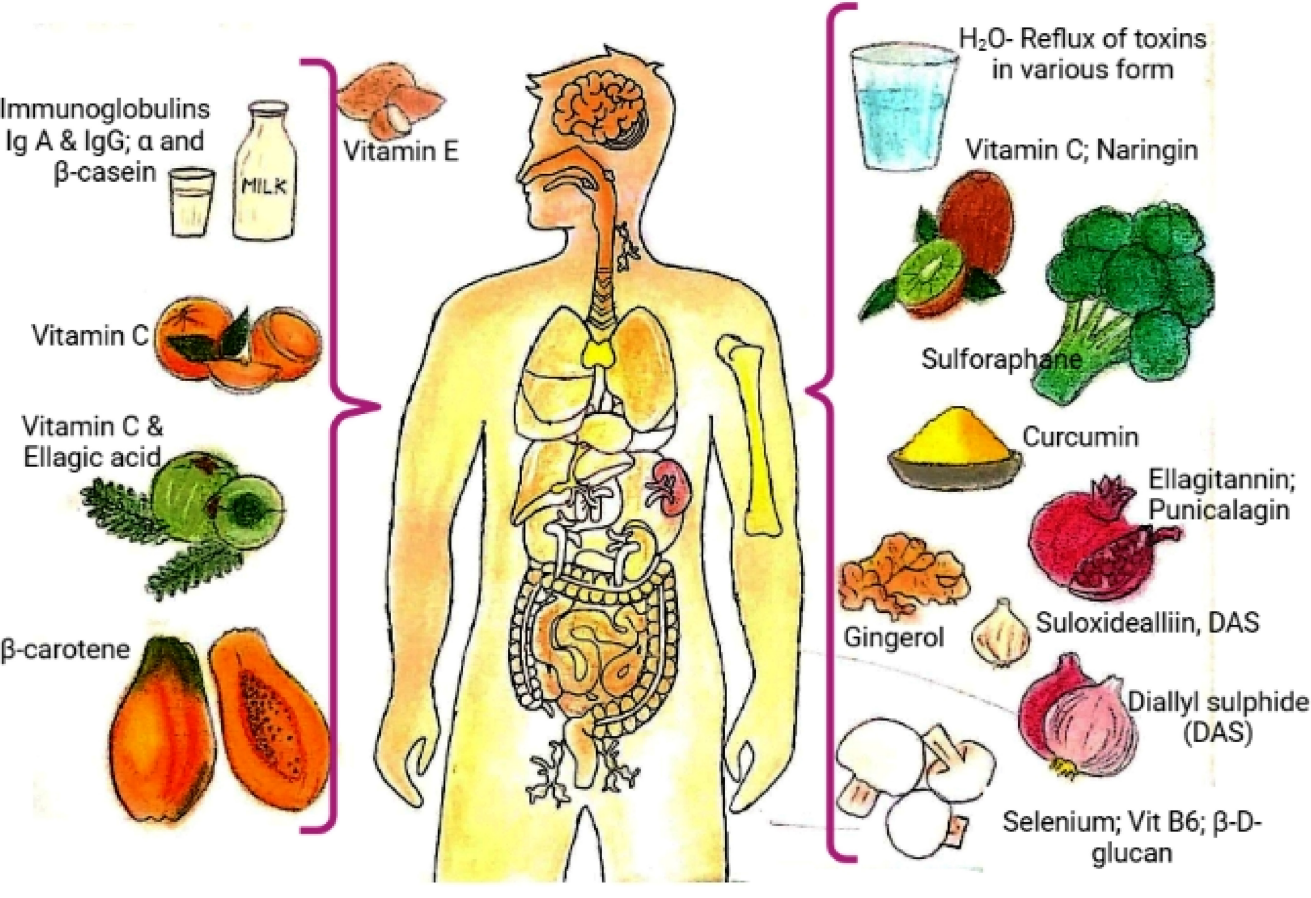
Bioactive compounds in various food items for boosting human immunity (Source: created by authors by taking inputs from Chowdhury & Barooah, 2020; Field et al., 2002; Hachimura et al., 2018; others as mentioned in Table 1).

With the COVID‐19 outbreak, Hippocrates' maxim “Let food be thy medicine, and medicine be thy food” becomes more vital than ever, as we need to find ways to enhance our immune system as much as possible. Making sure you eat a diet rich in immune‐boosting foods is the simplest method to stay healthy and fit. Nutrients are more readily absorbed by the human body when they are obtained from whole food sources such as fruits and vegetables rather than processed foods or supplements. Including a range of these foods and nutrients in your daily diet is more important than focusing on just one or two in big quantities. Proper blending of fruits such as papaya, citrus, pomegranate, etc., vegetables, spices, and animal‐based foods such as milk and eggs in the right proportions in daily diet has been shown to improve the functioning of the human immune system. These commonly available food items besides supplying carbohydrates, proteins, and energy to the body provide an array of bioactive compounds (Figure 2) which boost the immune system in various ways. We explored a range of foods rich in essential nutrients, antioxidants, and bioactive compounds that have been scientifically proven to strengthen the immune system. These include citrus fruits, berries, leafy greens, garlic, ginger, yogurt, nuts, and seeds, among others. Each of these foods offers unique benefits, from providing vitamin C and antioxidants to supporting gut health and reducing inflammation. It is important to note that a balanced and varied diet is crucial for overall health and immune system support. While incorporating immune‐boosting foods into our meals, we should also focus on maintaining a healthy lifestyle, including regular exercise, adequate sleep, and stress management. Furthermore, it is essential to consult with healthcare professionals or registered dietitians for personalized advice, especially if you have any specific dietary requirements or underlying health conditions.

By understanding the power of nutrition and making conscious choices about the foods we consume, we can proactively strengthen our immune system and promote our overall well‐being. Embracing a diet rich in immune‐boosting foods can be a delicious and enjoyable way to support our body's defense mechanisms, leading to a healthier and more resilient life.

## AUTHOR CONTRIBUTIONS

**Deo Narayan Singh:** Conceptualization (equal); data curation (equal); methodology (equal); writing – original draft (equal). **Jitendra Singh Bohra:** Supervision (equal); writing – review and editing (equal). **Tej Pratap Dubey:** Conceptualization (equal); formal analysis (equal); writing – original draft (equal). **Pushp Raj Shivahare:** Conceptualization (equal); formal analysis (equal); investigation (equal); validation (equal); writing – original draft (equal). **Ram Kumar Singh:** Resources (equal); visualization (equal); writing – review and editing (equal). **Tejbal Singh:** Conceptualization (equal); methodology (equal); writing – original draft (equal). **Deepak Kumar Jaiswal:** Conceptualization (equal); data curation (equal); formal analysis (equal); methodology (equal); writing – original draft (equal).

## FUNDING INFORMATION

The authors would like to declare that no external funding was received for the research, development, or publication of this article. The work presented herein is the result of independent efforts by the authors, who have not received any financial support or grants from any organization, institution, or individual.

## CONFLICT OF INTEREST STATEMENT

The authors declare that they have no financial or personal conflicts of interest that could influence the content or findings presented in this work. This includes but is not limited to any financial interests, affiliations, or involvements with organizations that may have a direct or indirect interest in the subject matter discussed in the manuscript.

## ETHICS STATEMENT

The preparation of this manuscript did not involve direct experimentation on any animals, including humans. Instead, this study relied on existing data, literature reviews, theoretical analyses, or other nonexperimental methodologies. As no living subjects, whether human or animal, were involved in this research, no ethical approval was required.

## Data Availability

Data sharing is not applicable to this article as no new data were created or analyzed in this study.
